# Disentangling the influence of living place and socioeconomic position on health services use among diabetes patients: A population-based study

**DOI:** 10.1371/journal.pone.0188295

**Published:** 2017-11-29

**Authors:** Sébastien Lamy, Denis Ducros, Chloé Diméglio, Hélène Colineaux, Romain Fantin, Eloïse Berger, Pascale Grosclaude, Cyrille Delpierre, Béatrice Bouhanick

**Affiliations:** 1 University of Toulouse III Paul Sabatier, Toulouse, France; 2 Department of Clinical Pharmacology, Toulouse University Hospital, Toulouse, France; 3 LEASP UMR1027 INSERM (The French National Institute of Health and Medical Research), Toulouse, France; 4 Agence Regionale de Santé (Regional Healthcare Agency), Occitanie, Toulouse, France; 5 Department of Epidemiology, Toulouse University Hospital, Toulouse, France; 6 Tarn Cancers Registry, Albi, France; 7 Institut Universtaire du Cancer de Toulouse–Oncopole, Toulouse, France; 8 Department of Hypertension and Therapeutics, Toulouse University Hospital, Toulouse, France; Weill Cornell Medical College in Qatar, QATAR

## Abstract

This research investigates the influence of place of residence and diabetic patient’s socioeconomic position on their use of health services in a universal health care system. This retrospective cross-sectional population-based study is based on the joint use of the Health Insurance information systems, an ecological indicator of social deprivation and an indicator of potential spatial accessibility of healthcare provision in the Midi-Pyrénées region. Using French healthcare insurance population-based data on reimbursement of out-of-hospital care during the year 2012, we study the use of health services among patients aged 50 and over (n = 90,136).We built logistic regression models linking health services use to socioeconomic position by geographic area, adjusted for age, gender, healthcare provision, information regarding patients precariousness, and long-term condition, used as proxy for the state of health. After adjustment for healthcare provision, the lower population density in the geographical area of concern, the lower the access to specialised care, independent of the patients’ SEP. General practitioner attendance was higher among the patients with the lowest SEP without being clearly influenced by their living place. We found no clear influence of either patients’ SEP or their living place on their access to biological follow-up. This study is an attempt to account for the geographical context and to go further in studying the social determinants of health among diabetes patients.

## Introduction

Many works support that socioeconomic-related differences exist in diabetes control and care, despite the presence of universal health coverage which may be seen as a facility that ensure the affordability of care. Patients with a lower socioeconomic position (SEP) had a lower rate of control of diabetes [1–3], lower access to preventive care as suggested by higher complication rates and morbidity [1, 4–8], higher general practitioner attendance rates but lower rates of hospital and specialised care attendance [1, 3, 4, 9–11]. These studies did not account for nor investigate the role of the geographical context despite evidences supporting the influence of the living place on patients’ use of health services, in different healthcare systems. Indeed, previous results from a study among elderly Medicare beneficiaries showed that patients from rural areas visited less often physician office, were hospitalised less often, but benefited from more home health visits than their counterparts from urban areas [12]. In the province of Quebec, a lower use of specialised services has been shown in non-metropolitan areas [13].

As suggested by Margolis et al. to explain strong geographical variation in the risk of lower-extremity amputation among Medicare beneficiaries with diabetes[14], we assume that health services provision, which depends on the living place, is not equally distributed in a given territory, as well as SEP. SEP and place of living refer respectively mainly to the financial affordability and the physical accessibility dimensions of access to care, conditioning health services use[15–17]. Although different, these dimensions are complementary in the understanding of what determines the use of health services. Thus, there is no doubt regarding the relevance of the assessment of the combined effect of SEP and place of living on patients’ use of the health services.

In France, the health coverage system is the same everywhere. Primary care is available to all resident and is delivered mainly in out-of-hospital settings. As for all patients with a chronic disease, the management of diabetes patients consists in delivering social and health services coordinated by a referent, mainly the general practitioner (GP). Once diabetes diagnosed, patients benefit from full healthcare coverage. A survey in 2007 shows that 95% of the population live at less than 15 minutes by the road from the nearest primary care service. Likewise, most of the GP and specialists in out-of-hospital are accessible to less than 20 minutes by road. However, inequalities in access persist. Areas with a low population density combine remoteness of both specialised and general care services [18]. In the present study, we will use data from the healthcare insurance on reimbursement of out-hospital care, healthcare provision and demographical databases to test the joint effect of SEP and place of residence on the way diabetes patients’ use of health services. We hypothesise that patients with the lowest SEP are more sensitive to the availability and accessibility of healthcare, especially to primary care.

## Methods

### Type of study and population

The design of this study was described in detail in a previous article that aimed to assess the socio-territorial determinants of access to healthcare[19]. Briefly, it is a cross-sectional study based on the joint use of the Health Insurance information systems, an ecological indicator of deprivation and an indicator of potential spatial accessibility of healthcare provision in the Midi-Pyrénées region. The French National Healthcare Insurance records prospectively reimbursement of out-of-hospital care. However, health services use data were merged with SEP and healthcare provision data and analysed retrospectively. According to the National Institute for Statistics and Economic Study, in 2012, there were approximately 2.9 million inhabitants in the region (about 5% of the whole French population) for a population density of 64.53 inhabitants per square kilometres. Midi-Pyrénées was the 8^th^ most populated French region but the 3^rd^ in terms of size with 45,347.9 square kilometres. A CNIL Authorisation (no. 1634837) was obtained for this study. This study included persons with the right to access, as of 31 December 2012, one of the three main health insurance schemes (General Regime (RG) from March 2012 to February 2013, Mutualité Sociale Agricole (MSA) and the Social Regime of Independents (RSI) for the year 2012). Some populations have been excluded due to differences in the management of the beneficiaries: the local mutual sections for the RG, the grouping of the health insurers of the operators for the MSA and self-employed professions for the RSI. After excluding 92,542 beneficiaries who died during the period of interest and 41,349 beneficiaries to whom no IRIS could be allocated, the base included 2,574,310 individuals, or about 87% of the total population of the region, of the same age and sex structure (data available on https://www.insee.fr/fr/statistiques/). In the case of the present study, given that type 2diabetes is predominantly in the population of patients with diabetes over the age of 50, we have restricted our analysis to individuals in this age group. Based on this database, we identified 957,911 beneficiaries aged 50 and over in the region, of whom 90,136 were considered to be diabetic, i.e. they had benefitted from pharmacy deliveries of at least three anti-diabetic medicines or insulin over the last year.

### Health services use markers

The quality of care for treated diabetic patients aged 50 years or older was assessed based on recommendations and by distinguishing between annual access to physicians (medical follow-up) and annual biological follow-up. For annual medical follow-up, we examined the proportion of patients who had at least three consultations with a general practitioner and the proportion of patients who received an ophthalmologic examination (at least a fundus or retinography or had a consultation with an ophthalmologist). For the annual biological follow-up, we studied the proportion of those who had at least three glycated haemoglobin assays, and the proportion of those who had at least one microalbuminuria assay. All of the indicators were calculated over 12 months.

### Collected variables

#### SEP

In the absence of individual social data, SEP was measured by an ecological deprivation index, the European Deprivation Index. The EDI approaches SEP by measuring social deprivation as defined by Townsend as a state of observable and demonstrable disadvantage relative to the local community or the wider society to which an individual, family or group belongs[20]. The EDI was calculated after geocoding the exact address of the person and assigning the grouped block for information (IRIS) corresponding to this address. IRIS is the smallest geographical unit for which statistics are available in France, which represents about 2,000 people. Each IRIS was assigned an EDI value, calculated from the 2007 census data. A high value signifies an IRIS with a high level of deprivation. We used an EDI presentation in deciles, calculated from all the IRISs of metropolitan France[21], from decile 1 corresponding to the least deprived zones to decile 10 corresponding to the most deprived zones. This index has already been used to study social inequalities in the access to healthcare in diabetic patients[22, 23]. In a paper published in the early 2017, Bryère et al. assessed the ecological bias by measuring the misclassification of individual SEP in seven ecological indices (Townsend index, Carstairs index, Lasbeur index, Havard index, the social (SCP) and material (MCP) components of Pampalon index, and the European Deprivation index (EDI)) used at the IRIS level [24]. They found that the aggregate indices studied were quite good “proxies” for SEP (Area Under the Curve close to 0.7), and they had similar performances. The indices were more efficient at measuring individual income than education or occupational category and are suitable for measuring of deprivation but not affluence. For each patient, information regarding precariousness was collected through whether or not the patient has access to French Supplementary Universal Healthcare Coverage (CMU-C) allocated under an income threshold giving entitlement, for patients with lower incomes, to cover an additional part of their healthcare expenses.

#### Healthcare needs

Patient healthcare needs, which may influence the level of access to healthcare, have been approached by variables recognised as determining these needs: age, sex and an exemption from co-payment due to long-term conditions, which is often used as a proxy variable of health status[25].

#### Healthcare provision

Healthcare provision was measured by potential localised accessibility (PLA) to a general practitioner[9].It assesses the availability and proximity of healthcare, two dimensions characteristic of spatial access to healthcare at the township-level [26]. PLA was developed in France by the Department of Research, Studies, Evaluation and Statistics (DRESS), using North American works in particular[27].The method of calculating the PLA is presented in detail elsewhere[19]. Briefly, it considers a general practitioner’s level of activity, in full-time equivalence, to measure the distance-weighted supply and the age-differentiated rate of access to measure demand. For the present work, it has been calculated at the IRIS level considering the supply of doctors and the demand in the surrounding IRIS. It is interpreted as a medical density in number of full-time equivalents per 100,000 inhabitants. The PLA of self-employed general practitioners was calculated on 1 January 2013. The distance by road was calculated between medical analysis laboratories and the residential IRIS. We assumed that the supply of doctor can be saturated by the demand whereas the medical analysis laboratories cannot.

#### Geographic area

**Fig 1:**
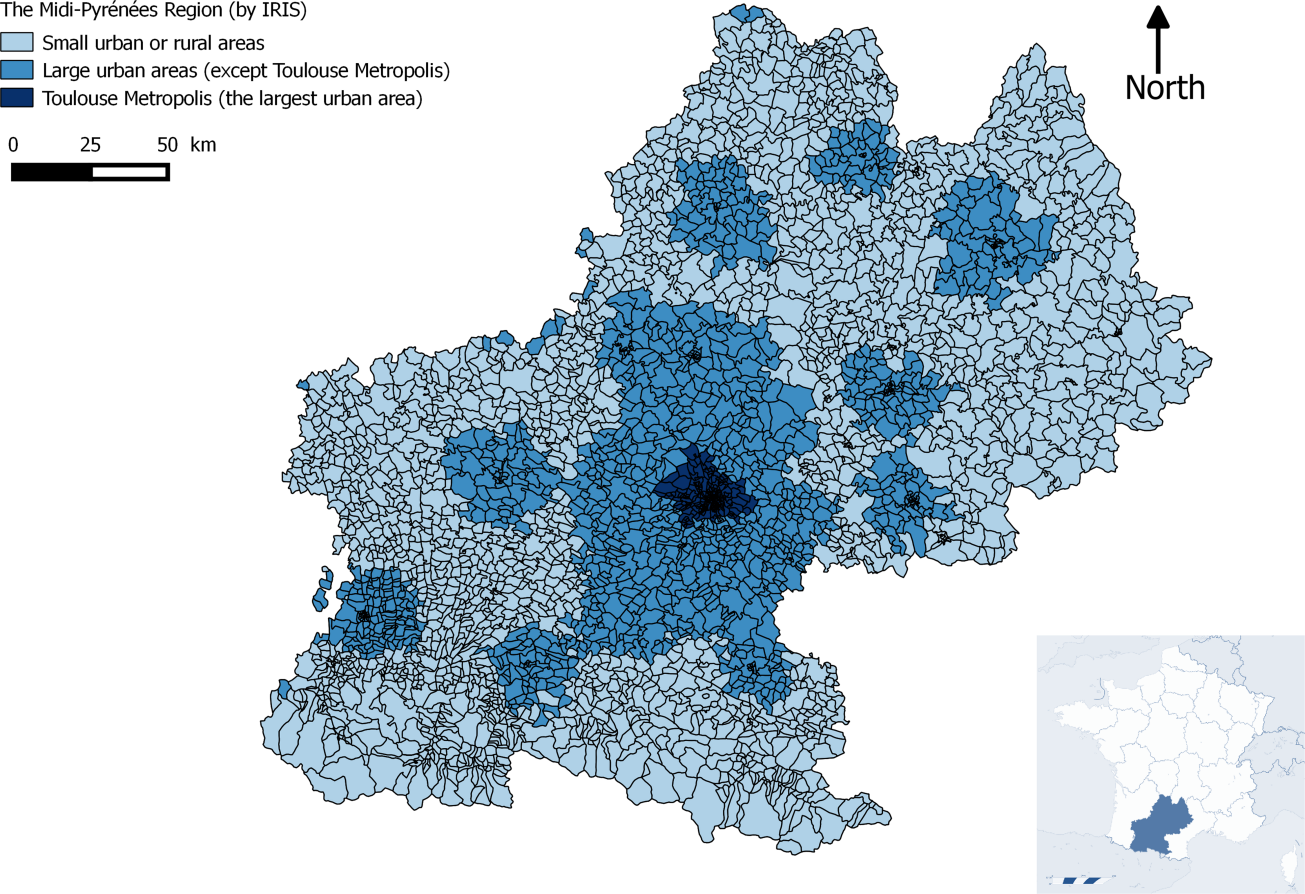
The level of urbanisation of the IRIS of the Midi-Pyrénées region.

In 2015, Toivakka et al supported the interest of studying geographical context on the assessment of control and treatment targets among type 2 diabetes patients using areas classification that outreach the urban-rural dichotomy based on municipal borders[28]. In the present study, we assumed that both the health services and transport facilities increased with the level of urbanization. To take into account the potential differences in the organisation of healthcare provision according to the level of urbanisation of the region, we used the French National Institute of Statistics and Economic Studies (INSEE)’s 2010 zoning in urban areas, which corresponds to the areas of influence of cities and their agglomerations in the region, beyond their physical limits. We distinguished between the large urban areas, defined by an entire large urban hub (more than 10,000 jobs) and its suburbs, from the rest of the region. Among the large urban areas, we were also more specifically interested in the Toulouse metropolis, which is the main urban area of the region with almost one fifth of the region's population living there (see Fig 1).

### Statistical analysis

To investigate the role of region and SEP, we constructed a ten-modal by geographical area indicator corresponding to the EDI deciles of each type of region (T1 to T10 for the Toulouse metropolis, U1 to U10 for other large urban areas, and R1 to R10 for the rest of the region). We used logistic regression models linking each indicator of health services use to EDI by geographic area, adjusted for age, gender, and whether the patient benefits from CMU-C and has an exemption from co-payment due to long-term condition, used as proxy for the state of health, healthcare provision evaluated by PLA when supply is saturated and evaluated by access time in the event of unsaturated supply. The adjusted odds-ratios (ORs) associated with each decile of the EDI of each type of geographical area were represented in graphical form using the most privileged EDI decile (highest SEP) of the Toulouse metropolis as reference. Statistical analyses were conducted using STATA software version 14 (StataCorp LP, College Station, TX, USA).

## Results

**Fig 2:**
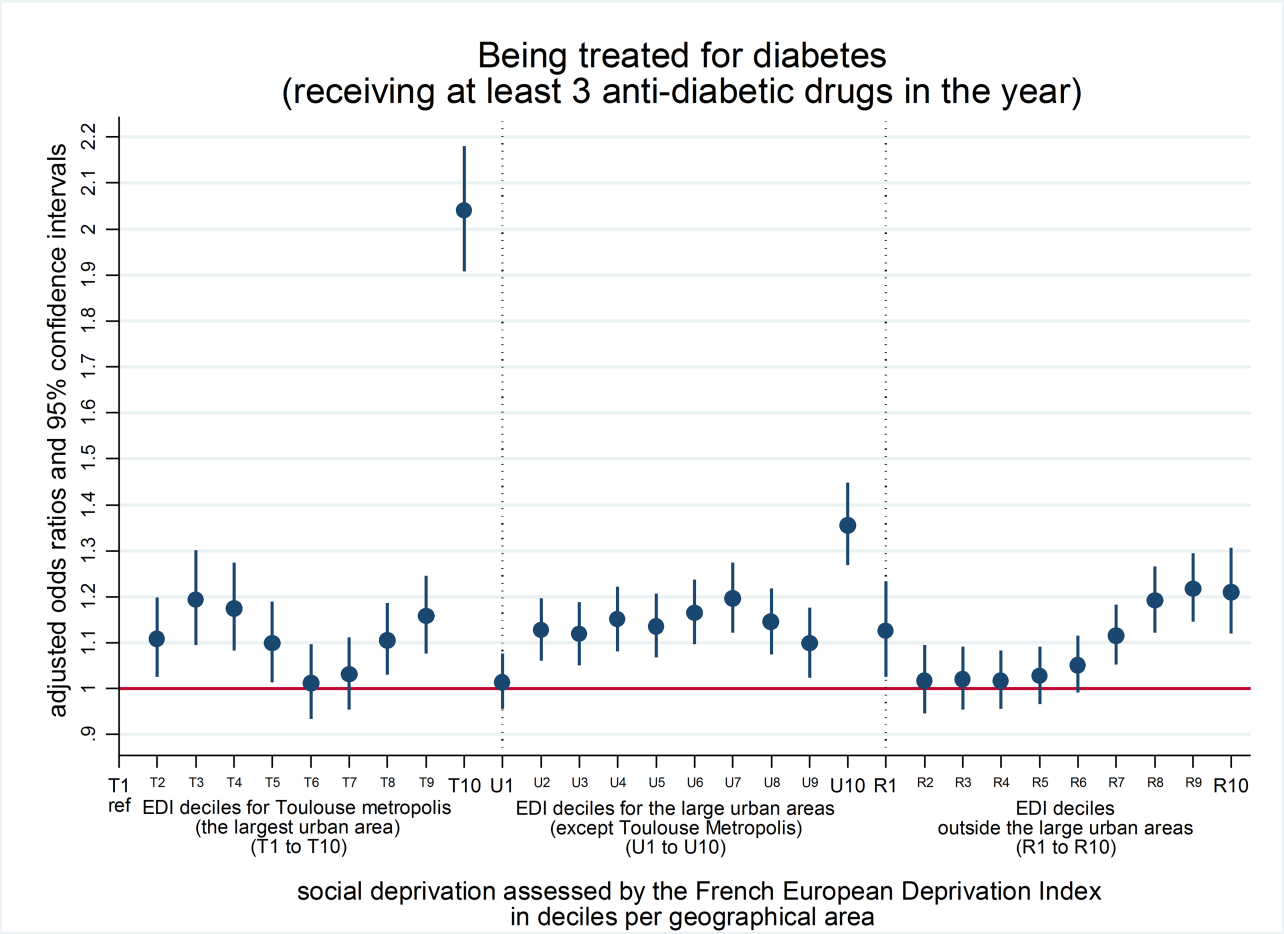
Being treated for diabetes (i.e. intake of at least three anti-diabetic drugs during the year) by SEP and living place among individuals aged 50 or over in the Midi-Pyrénées region (n = 957,911). Results from a logistic model adjusted for SEP by geographical area, age, sex, exemption from co-payment due to long-term condition.

Of the 957,911 individuals aged 50 years or older identified in the database, 90,136 were considered to be diabetic, representing 9.1%, 9.3% and 9.6%, of people living in the Toulouse metropolitan area, in other major urban areas, and in the rest of the region respectively. The proportion of diabetic individuals, i.e. of individuals receiving at least 3 anti-diabetic drugs during the year, was higher among the most socially disadvantaged, with only a modest influence on the type of region of residence (Fig 2).

Among patients aged 50 years or older treated for diabetes, we can see that those living in large urban areas were younger, more frequently disadvantaged, more often beneficiaries of CMU-C and less often had a long-term condition than those living outside large urban areas. These trends were even more marked inpatients in the Toulouse metropolis compared to those living outside large urban areas (Table 1).

**Table 1:** The characteristics of the 90,136 patients aged 50 or over and treated for diabetes in the Midi-Pyrénées region.

		Toulouse metropolis^1^		others large urban areas		Midi-Pyrénées region except the large urban areas	
		(n = 16,412)		(n = 39,048)		(n = 34,676)	
		n	%	n	%	n	%
**age**	**mean (±sd)**	70	±10.6	71	±10.5	72	±10.3
**social status (European deprivation index in decile)**	**1 –most privileged**	1,872	11.4	4,847	12.4	840	2.4
	**2**	1,425	8.7	4,662	11.9	1,746	5.0
	**3**	1,026	6.3	4,088	10.5	2,455	7.1
	**4**	1,240	7.6	4,282	11.0	3,687	10.6
	**5**	1,247	7.6	4,450	11.4	4,224	12.2
	**6**	1,233	7.5	4,523	11.6	5,557	16.0
	**7**	1,449	8.8	3,313	8.5	5,785	16.7
	**8**	2,102	12.8	3,633	9.3	4,653	13.4
	**9**	1,749	10.7	2,261	5.8	4,232	12.2
	**10 –most disadvantaged**	3,069	18.7	2,989	7.7	1,497	4.3
**sex**	**Men**	8,734	53.2	21,178	54.2	18,712	54.0
	**Women**	7,678	46.8	17,870	45.8	15,964	46.0
**CMU-C**	**No**	15,278	93.1	37923	97.1	33,876	97.7
	**yes**	1,134	6.9	1,125	2.9	800	2.3
**exemption of co-payment due to long term condition**	**No**	2,865	17.5	6,101	15.6	5,048	14.6
	**Yes**	13,547	82.5	32,947	84.4	29,628	85.4
**potentially localised accessibility (med (IQR**^**2**^**))**	**to a GP**	104	45	75	43	79	51
	**to an ophthalmologist**	12	3	6	3	3	3
**distance in time (in minutes), from the nearest medical laboratory (med (IQR))**		2	2	5	7	10	14

^1^ Toulouse metropolis encompasses 37 municipalities. It corresponds to the most significant large urban areas of the region.

^2^ med (IQR) stands for median with interquartile range (IQR = q3 –q1)

**Fig 3:**
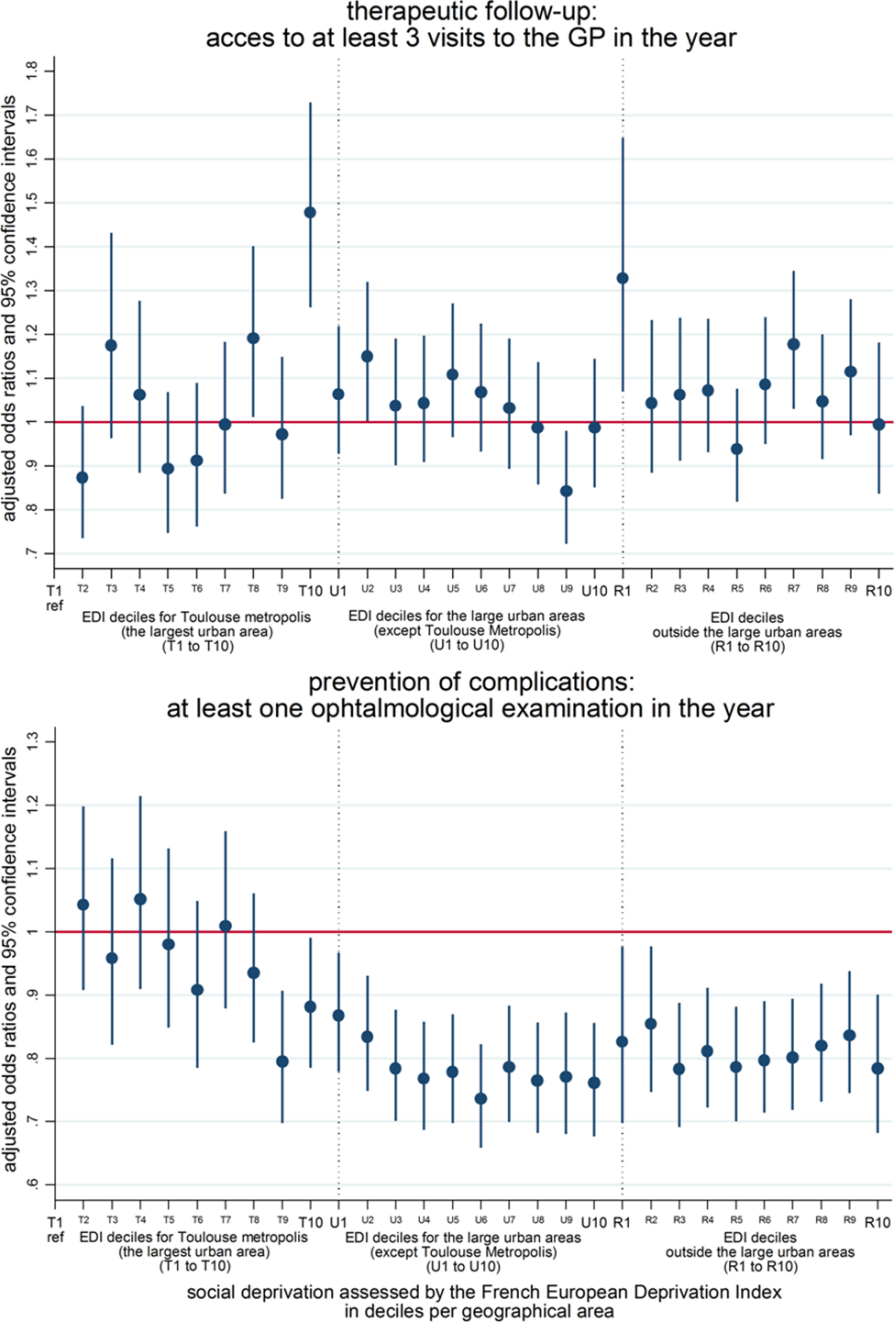
Access to medical follow-up by SEP and living place among patients aged 50 or over treated for diabetes. **(n = 90,136).** Results from a logistic model adjusted for SEP by geographical area, age, sex, exemption from co-payment due to long-term condition, universal complementary healthcare insurance, and potentially localised accessibility to the GP (for therapeutic follow-up) / ophthalmologist (for prevention of complications).

The results of the analysis of the association between the level of a patient’s SEP by type of region and the medical follow-up are presented in Fig 3 for the follow-up by general practitioners and prevention of ophthalmological complications. Overall, no association between access to a general practitioner and a patient’s SEP or the type of their region of residence was found, except for a higher access rate among the most disadvantaged patients in the Toulouse metropolis, as well as among the most privileged patients in the least urbanised region of the territory Regarding the prevention of complications, differences according to the patient’s type of region of residence were found but not according to their SEP except in Toulouse metropolis where the most disadvantaged patients had lower access rates. The access to at least one annual ophthalmologic examination was lower in patients living outside the Toulouse metropolis than in those living in this area, irrespective of SEP (Fig 3).

**Fig 4:**
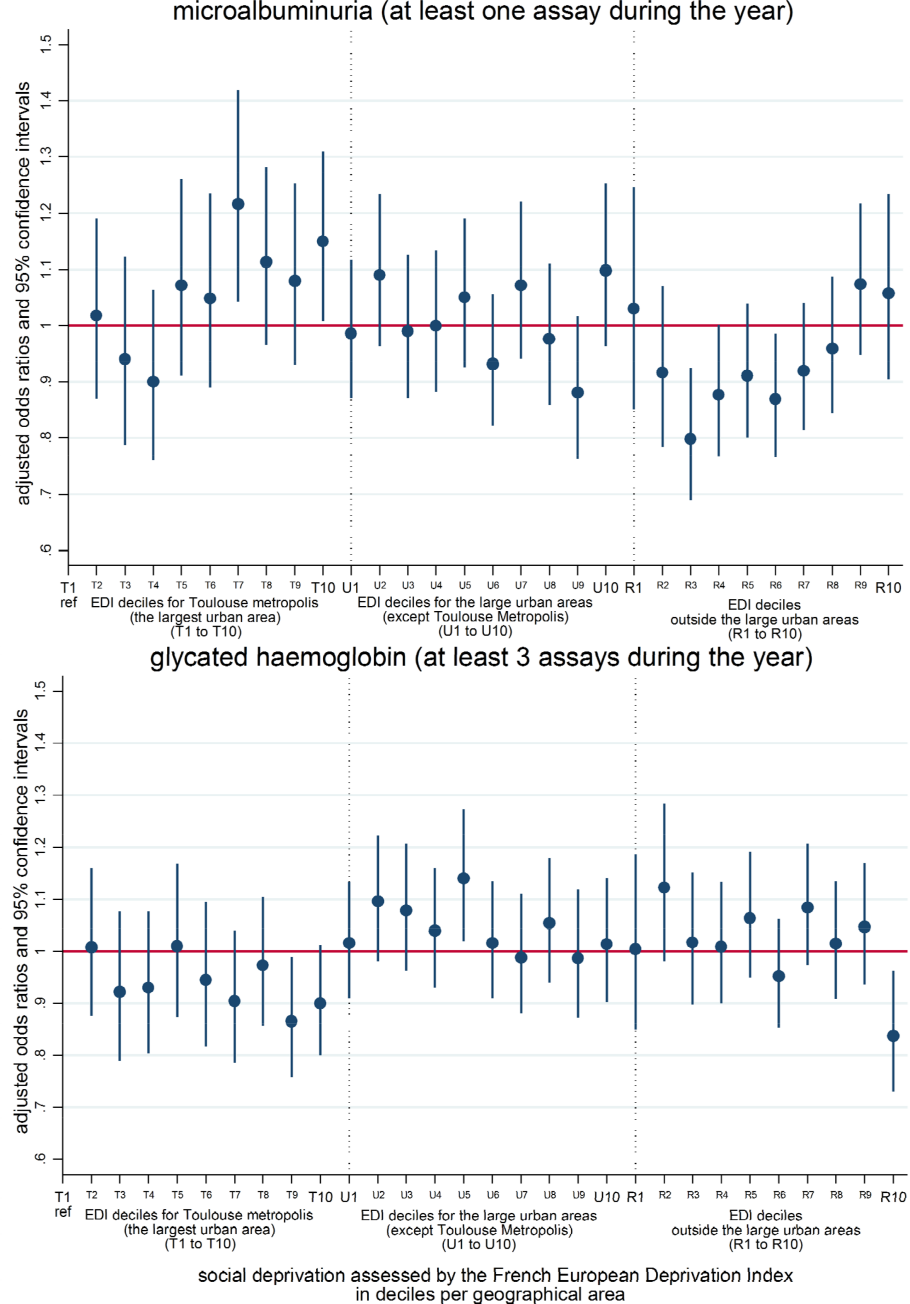
Access to biological monitoring (microalbuminura and glycated haemoglobin) by SEP and living place among patients aged 50 or over treated for diabetes. **(n = 90,136).** Results from a logistic model adjusted for SEP by geographical area, age, sex, exemption from co-payment due to long-term condition, universal complementary healthcare insurance, and distance from the nearest medical laboratory.

Finally, results from patients’ biological follow-up show no clear social gradient or influence of geographical area of residence on access to at least three glycated haemoglobin assays as well as access to at least one microalbuminuria assay in Fig 4. However, regarding the glycated haemoglobin monitoring, a lower access rate was found among the most disadvantaged patients in the territory outside the large urban areas and in Toulouse metropolis in a lesser extent. Regarding the microalbuminuria monitoring, patients with an intermediate SEP in the Toulouse metropolis and the most privileged patients outside the large urban areas tended to have respectively higher and lower access rates.

## Discussion

This work aimed at studying the influence that patients’ living place and SEP have on their use of health services. Our results show that a patient’s region of residence has an influence on medical follow-up by a specialist, independent of the patient’s SEP, assessed by an ecological deprivation index. Indeed, access to at least one ophthalmological examination during the year was lower outside than inside the Toulouse metropolis where the most disadvantaged patients had lower access rates. A lower influence of the geographical context appeared for the probability of being diabetic and for access to a GP. We observed higher rates of diabetic patients among people with the lowest SEP and more importantly, the size of this effect diminished from the most densely (Toulouse metropolis) to the least densely (outside large urban areas) populated region. Regarding access to a GP, higher rates were observed outside the urban areas among patients with highest SEP and in the Toulouse metropolis among patients lowest SEP. In the other urban areas, patients with the second lowest SEP presented the lowest access to a GP. Our results concerning the biological follow-up of glycated haemoglobin and microalbuminuria showed no clear differences related to either SEP or geographical areas.

The present study complements existing literature on the socioeconomic-related [1–6, 9, 10] and geographical differences in the way diabetic patients’ are cared for and their use of the health system in a universal health coverage setting [7, 12–14]. Regarding the French situation, the results of the national surveys on a representative sample of people with diabetes (ENTRED) carried out in 2001–2003 and 2007–2010 produced similar conclusions. Patients with low SEP were more often diagnosed late, had more complications, were more frequently followed up by general practitioners and did not visit endocrinologists, ophthalmologists and dentists as often as those with a higher SEP[3]. These results were based on the use of individual socioeconomic data, measured by a self-administered questionnaire, resulting in high rates of missing data. In order to overcome this limitation, work was carried out using socioeconomic data collected on a higher scale, using ecological indicators of SEP, to approach an individual’s SEP. Thus using such ecological measures of SEP, the differences in patient care according to SEP already observed in the previous studies remained stable or even decreased between 2001 and 2007, except for the use of specialist doctors for whom the improvement mainly concerned patients from more privileged social groups[23]. For the period 2010–2013, social disparities were low regarding biological follow-up but high regarding therapeutic follow-up and the occurrence of complications [29]. To go further, in the present study, we account for the interaction between a patient’s SEP and the type of region where they live. Moreover, our study allows us to disentangle the respective influence of these factors on patient use of health services. Indeed in this study, we found that the effect of a patient’s SEP on general practitioner attendance rates depended on where people lived: they were higher among the most deprived patients in the Toulouse metropolis area but among the least deprived patients outside the large urban areas. These observations may translate to the fact that in the most urbanised areas where both the general and specialised services provision is high, the patients with a higher SEP use specialised services more often in contrast to those with a lower SEP who use the general services more often. This inequality in the use of specialised services is well known and observed in many countries [30, 31].Outside large urban areas, the general services provision, although lower than in more urbanised areas, is much higher than those of the specialised services. The higher general services attendance rate observed among patients with the highest SEP may simply translate a better access to the available healthcare services. Regarding the attendance of specialised services, our results on access to an ophthalmological examination shows no influence of a patient’s SEP, assessed by an ecological deprivation index. However, we found that geographical context has a strong influence as patients from outside large urban areas had lower access to ophthalmological examinations that falls within the competence of specialist doctors. As our models were adjusted for ophthalmological services accessibility approached by the potentially localised accessibility, the geographical influence we found may reflect something different than the effect of the provision and accessibility of such services. We assume that this may be a result of the distance from a patient’s residence to specialised services whatever they are available or not. Here the distance in itself and what patients have to do to face it might act as a barrier to the use of specialised services in this settings. Another explanation might be that, outside large urban areas there is a shortage of supply affecting everyone, i.e. there is not enough supply to allow for SEP-related variations in its use. Lastly, we have to temper our results showing that neither a patient’s SEP nor the geographical context in which they live has any clear influence on biological follow-up. We have no information on the biological values and recent national observations show that, although the biological follow-up of diabetics has improved during the last decade, it was still weak in 2013 with 30% and 51% of patients with at least one microalbuminuria assay and 3 HbA1c assays over the year respectively [29]. This was still far from the objective of 80% in the 2004 Health Insurance Act.

The major limitation of this study is that it is based on the use of administrative data that only encompasses the reimbursement of out-of-hospital care. Due to this we may miss many of the acts that may have occurred in a hospital setting. Some previous results among Medicare diabetic patients showed that rural beneficiaries used fewer hospital days and physician office visits but more home health visits than their urban counterparts [12]. Thus, in our study, we might have underestimated the influence of the geographical context due to the unavailability of in-hospital care which is assumed to be more frequent among urban patients. However, this is lessened by the fact that we focused on indicators of healthcare system use for which there are little to no in-hospital substitutes on offer. In addition, in our study, diabetes was defined from the identification of at least three anti-diabetic drugs during the year which means that we focused on patients that were already treated for their diabetes, that is, people that are already in the process of receiving care. The consequence of that is that we may again underestimate the influence of the geographical context and a patient’s SEP. Indeed, we automatically exclude people with no treatment or medical follow-up and who may likely have a lower social status or who are more likely to live in deprived areas [10, 32, 33]. Finally, this study used regional data which may limit the generalisation of our observation. However, it is lessened by the fact that the healthcare coverage system is national, i.e. the same everywhere in the French territory. It should be noted, in contrast, that we had access to a large sample representing almost 90% of the whole population of the region of interest [22]. We found no difference in the age and sex structures between our sample and the whole regional population from the data yielded by the National Institute for Health and Economics Study for the same year (data available on https://www.insee.fr/fr/statistiques/)). Moreover, by compiling data from several sources we could account for patients’ SEP, even if we used an ecological-level index as a proxy of the individual’s status, and patients’ environment in terms of healthcare provision and geographical context. This allowed us to relax the assumption tacitly made in previous studies on social inequalities in health among the general population of French diabetic patients which was that a patient’s SEP had the same effect independent of the geographical context in which they live[3]. Lastly, although we had access to data combining both the patients’ SEP and the geographic characteristics of their living areas, we used specific indicators as proxy of multidimensional concepts as SEP and the spatial accessibility to health care provision. Thus, we may have only a partial vision of the phenomena linking these factors to a diabetic patients’ use of health services. Future works would benefit from a more comprehensive approach of these concepts especially when addressing regional health disparities which may imply a combination of several factors such as culture, lack of opportunities and regional organisation [34].

## Conclusion

This study shows the importance of contextualising the study of the social determinants of diabetic patients’ use of health services. Indeed, we show that ignoring the influence of the geographical context in which patient lives may lead to the partial and incorrect interpretation of the role some well-established determinants of diabetic patients’ play in the use of health services, such as their SEP. This would not be possible without merging data from different sources to adopt a comprehensive approach of the problem.
